# Race- and Ethnicity-Related Differences in Heart Failure With Preserved Ejection Fraction Using Natural Language Processing

**DOI:** 10.1016/j.jacadv.2024.101064

**Published:** 2024-07-02

**Authors:** Sam Brown, Dhruva Biswas, Jack Wu, Matthew Ryan, Brett S. Bernstein, Natalie Fairhurst, George Kaye, Ranu Baral, Antonio Cannata, Tom Searle, Narbeh Melikian, Daniel Sado, Thomas F. Lüscher, James Teo, Richard Dobson, Daniel I. Bromage, Theresa A. McDonagh, Ali Vazir, Ajay M. Shah, Kevin O’Gallagher

**Affiliations:** a King's College Hospital NHS Foundation Trust, London, United Kingdom; b School of Cardiovascular and Metabolic Medicine & Sciences, British Heart Foundation Centre of Research Excellence, King's College London, London, United Kingdom; c Institute of Psychiatry, Psychology and Neuroscience, King's College London, London, United Kingdom; d Royal Brompton and Harefield Hospitals, Guy's and St Thomas' NHS Foundation Trust, London, United Kingdom

**Keywords:** AI (artificial intelligence), health equity, heart failure, Natural Language Processing, preserved ejection fraction

## Abstract

**Background:**

Heart failure with preserved ejection fraction (HFpEF) is the predominant form of HF in older adults. It represents a heterogenous clinical syndrome that is less well understood across different ethnicities.

**Objectives:**

This study aimed to compare the clinical presentation and assess the diagnostic performance of existing HFpEF diagnostic tools between ethnic groups.

**Methods:**

A validated Natural Language Processing (NLP) algorithm was applied to the electronic health records of a large London hospital to identify patients meeting the European Society of Cardiology criteria for a diagnosis of HFpEF. NLP extracted patient demographics (including self-reported ethnicity and socioeconomic status), comorbidities, investigation results (N-terminal pro-B-type natriuretic peptide, H_2_FPEF scores, and echocardiogram reports), and mortality. Analyses were stratified by ethnicity and adjusted for socioeconomic status.

**Results:**

Our cohort consisted of 1,261 (64%) White, 578 (29%) Black, and 134 (7%) Asian patients meeting the European Society of Cardiology HFpEF diagnostic criteria. Compared to White patients, Black patients were younger at diagnosis and more likely to have metabolic comorbidities (obesity, diabetes, and hypertension) but less likely to have atrial fibrillation (30% vs 13%; *P* < 0.001). Black patients had lower N-terminal pro-B-type natriuretic peptide levels and a lower frequency of H_2_FPEF scores ≥6, indicative of likely HFpEF (26% vs 44%; *P* < 0.0001).

**Conclusions:**

Leveraging an NLP-based artificial intelligence approach to quantify health inequities in HFpEF diagnosis, we discovered that established markers systematically underdiagnose HFpEF in Black patients, possibly due to differences in the underlying comorbidity patterns. Clinicians should be aware of these limitations and its implications for treatment and trial recruitment.

Heart failure with preserved ejection fraction (HFpEF) accounts for approximately half of all cases of heart failure (HF),^1^^,^^2^ with an increasing prevalence among older adults and ethnic minority groups.^3^ HFpEF is considered to be a clinically heterogenous syndrome, posing a diagnostic challenge.^1^ To address this challenge, the H_2_FPEF score was developed to aid diagnosis and has subsequently demonstrated prognostic power.^4^, ^5^, ^6^, ^7^ However, its applicability across diverse ethnicities has yielded mixed results^8^; in a cohort of 233 Asian adults with a clinical diagnosis of HF and left ventricular ejection fraction (LVEF) ≥50%, the H_2_FPEF score had a sensitivity of 24.9%.^8^ Health equity in HFpEF management requires diagnostic tools with high efficacy in all patient groups. This clinical need has increasing urgency with recent trial data demonstrating prognostic benefit in HFpEF treated with sodium glucose cotransporter-2 inhibitors (SGLT2i).^9^^,^^10^

A distinct concern arises in the disproportionate impact of HFpEF on Black patients, who are affected at a younger age, possibly related to their higher prevalence of key HFpEF risk factors including hypertension, obesity, and diabetes.^3^ However, despite these disparities, HFpEF research and trials have predominantly investigated White populations.^7^^,^^11^^,^^12^ Thus, less is known about the relationship between race/ethnicity and HFpEF, with resultant potential for health inequity.

We applied a validated Natural Language Processing (NLP) algorithm to the electronic health records (EHRs) of a large central London secondary and tertiary care hospital, to identify all HF cases that fulfilled the full European Society of Cardiology (ESC) 2021 HFpEF diagnostic criteria.^13^ This novel approach, using artificial intelligence (AI) to extract undiagnosed HFpEF cases from the free-text portion of EHR, thereby acquiring a more detailed clinical picture than that from structured data and aiming to mitigate bias associated with a clinician assigned diagnosis, including the scale and harms of undiagnosed HFpEF as outlined in our recent study.^14^ Here, we utilize this cohort of HFpEF patients to examine racial and ethnic disparities in the performance of existing diagnostic markers (H_2_FPEF score and N-terminal pro-B-type natriuretic peptide [NT-proBNP]) and linking these to underlying comorbidities and survival outcomes.

## Methods

### Study cohort

**Figure 1:**
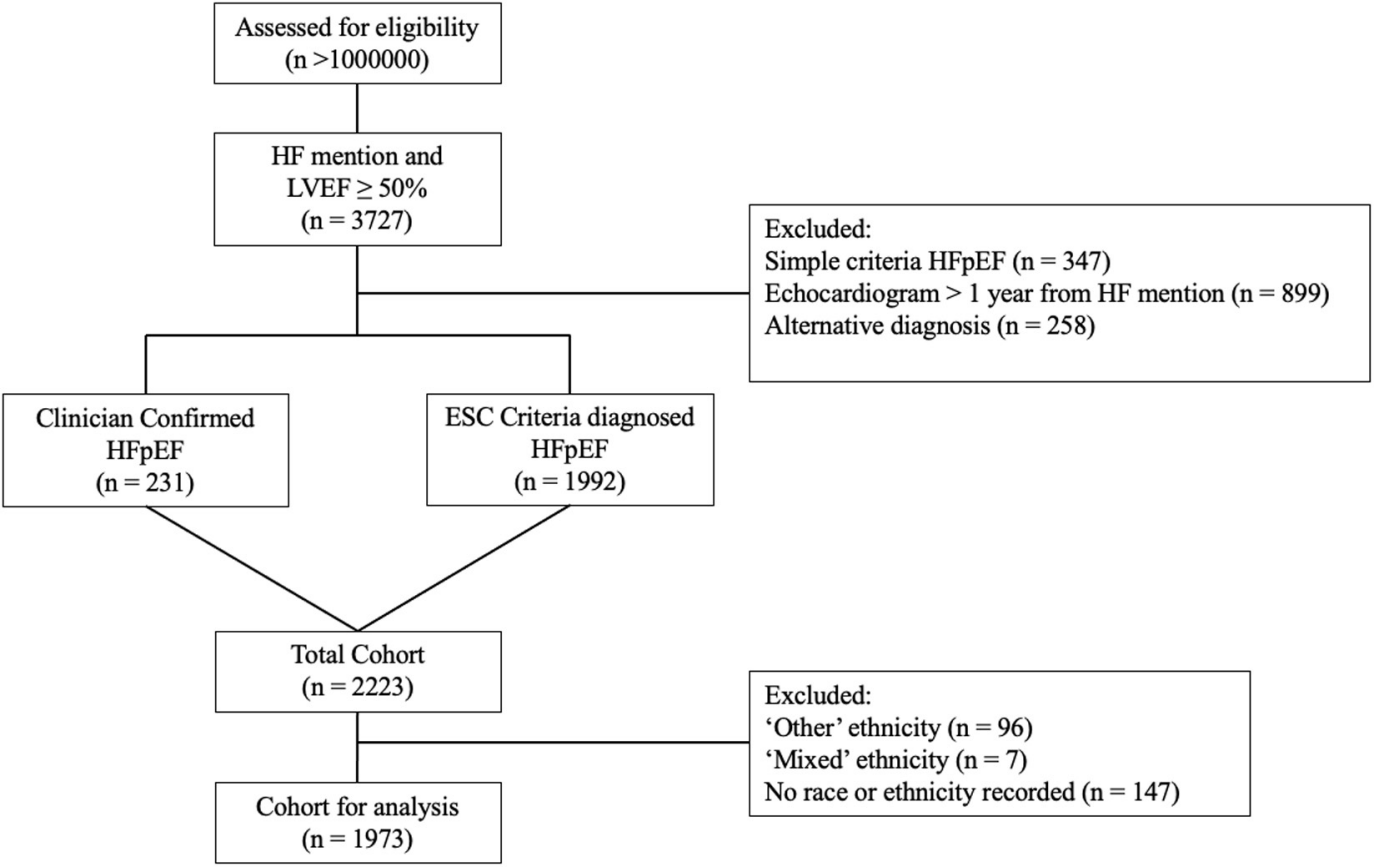
**Cohort Overview** CONSORT (Consolidated Standards of Reporting Trails) diagram of patient inclusion and exclusion criteria. Simple criteria HFpEF includes patients with clinical signs and symptoms of HF and LVEF ≥50% but do not meet the additional structural and/or biochemical ESC HFpEF criteria for inclusion. ESC = European Society of Cardiology; HF = heart failure; HFpEF = heart failure with preserved ejection fraction; LVEF = left ventricular ejection fraction.

An NLP tool was used to establish a single-center retrospective database of adult patients with a diagnosis of HFpEF from EHR at King’s College Hospital National Health Service Foundation Trust between 2010 and 2022, as detailed in our recent study.^14^ The study operated under London South-East Research Ethics Committee approval granted to the King's Electronic Records Research Interface, which did not require written informed patient consent. This study complies with the Declaration of Helsinki. Briefly, patients were included, whether inpatient or outpatient, if there were two or more mentions of a diagnosis of “heart failure” in the clinical notes determined by NLP.^14^^,^^15^ Next, variables were extracted from echocardiogram reports. Patients were only included for assessment if they had a LVEF ≥50% recorded within a year of the first HF mention and excluded if at any point their LVEF was <50%. Patients were also excluded if there was an alternative diagnosis documented including hypertrophic cardiomyopathy, restrictive cardiomyopathy, constrictive pericarditis, cardiac amyloidosis, or severe valvular disease. Patients were included if they met the ESC criteria for a diagnosis of HFpEF. This includes clinical signs and symptoms of HF (as indicated above by “heart failure” in the clinical notes), an LVEF ≥50%, and “evidence of cardiac structural and/or functional abnormalities consistent with the presence of LV diastolic dysfunction/raised LV filling pressures”^13^ (Figure 1). The latter includes one of: raised NT-proBNP ≥125 pg/mL (≥375 pg/mL with atrial fibrillation [AF]), LV mass index ≥95 g/m^2^ (female) 115 g/m^2^ (male), relative wall thickness >0.42, left atrial volume index >34 mL/m^2^ (>40 mL/m^2^ with AF), Doppler echocardiographic E/e’ (ratio of early diastolic mitral inflow velocity to mitral annulus relaxation velocity) ratio >9, pulmonary artery systolic pressure (PASP) >35 mm Hg, or tricuspid regurgitation velocity >2.8 m/s.^13^ This methodology allowed us to identify patients who met the ESC criteria even if they have not been given a formal diagnosis of HFpEF (“Undiagnosed HFpEF”). “Confirmed HFpEF” or a clinician-assigned diagnosis of HFpEF was identified from NLP using free-text documentation of “HFpEF” mentions in the clinical notes. For confidence in the NLP-based retrieval, manual validation of the presence of “HFpEF” mentions was performed on 100 randomly sampled (49 of whom were White, 35 were Black, 10 were Asian, and 5 did not have a recorded ethnicity) NLP identified clinician-assigned HFpEF patients (ie, “Confirmed HFpEF”) with a 100% true positive rate.

### Data processing

SNOMED-CT (Systematized Medical Nomenclature for Medicine–Clinical Terminology) concepts were extracted from EHR using the validated MedCAT (Medical Concept Annotation Tool)^16^ and MedCATTrainer^17^ NLP tools available from the CogStack ecosystem. NLP extraction and data processing have been detailed in our recent study.^14^ The performance of the MedCAT NLP pipeline in detecting comorbidity mentions has been previously assessed on 5,617 annotations from 265 documents, and the F1 (harmonic mean of precision and recall) measures were all >0.85.^18^ External validation of the cohort has been previously performed and demonstrated a similar pattern of clinical characteristics and mortality differences between the Confirmed HFpEF and Undiagnosed HFpEF cohorts.^14^

### Study variables

The SNOMED-CT concepts extracted from the clinical notes included demographics (age, sex), comorbidities (diabetes mellitus type 2, hypertension, chronic kidney disease [CKD], AF, myocardial infarction, coronary artery disease [CAD], stroke, and transient ischemic attack), medications (calcium channel blockers, beta-blockers, loop diuretics, mineralocorticoid receptor antagonists, angiotensin-converting enzyme inhibitors, angiotensin receptor blockers, SGLT2i, and insulin), and laboratory values (NTproBNP, BNP, hemoglobin, creatinine, HbA1c, and body mass index [BMI]). Self-reported race and ethnicity were obtained from clinical coded data. When reporting race and ethnicity, we adhered to the latest scientific guidance on their reporting.^19^ Only terms predating the first HF mention were included as comorbidities. Baseline characteristics were reported according to race and ethnicity groups. Hospitalizations were determined from discharge summaries. Postcodes were cross referenced with The English Indices of Deprivation 2019 statistics to calculate an Index of Multiple Deprivation (IMD) score as a surrogate for socioeconomic status.^20^ Ethnicities were compared according to the proportion in the lowest IMD quintile. H_2_FPEF scores (0-9) were calculated and stratified into low (scores 0-1), intermediate (scores 2-5), and high scores (scores 6-9, considered diagnostic of HFpEF).^7^^,^^21^ The score is made up of a BMI >30 kg/m^2^ (2 points), hypertension (1 point), AF (3 points), PASP >35 mm Hg (1 point), age >60 years (1 point), and E/e’ ratio >9 (1 point).^7^

### Outcomes

The primary end point was all-cause mortality. Mortality data were obtained from death notification letters in EHR system. Stroke and new-onset AF were recorded as outcome data if they occurred after the first mention of HF. Hospitalizations were determined from discharge summaries.

### Statistical analysis

The risk of all-cause mortality was assessed using Kaplan-Meier estimates and log-rank values to compare ethnicity groups. Survival curves were plotted for the following subgroups: NT-proBNP levels ≥125 pg/mL and H_2_FPEF scores ≥6, and ethnicities compared using log-rank tests. Baseline continuous variables were summarized as mean ± SD, or median (IQR); categorical data were summarized as counts and percentages. Continuous variables were compared with a *t*-test or Kruskal–Wallis test; categorical variables were compared with a chi-square test. A Cox regression analysis was performed to compare survival between ethnicities adjusted for age, sex, IMD score, NT-proBNP, and diagnostic group. As the largest patient subgroup, White patients were used as the reference ethnicity group. The results of the Cox regression analysis are presented as HRs with corresponding 95% CIs. A separate Cox regression analysis was also performed on each ethnicity subgroup to test the association between H_2_FPEF score and NT-proBNP with mortality. *P* values <0.05 were designated statistically significant. All analyses were performed using R Statistical Software (version 4.3.1; R Core Team 2024).

## Results

### Patient demographics

**Figure 2:**
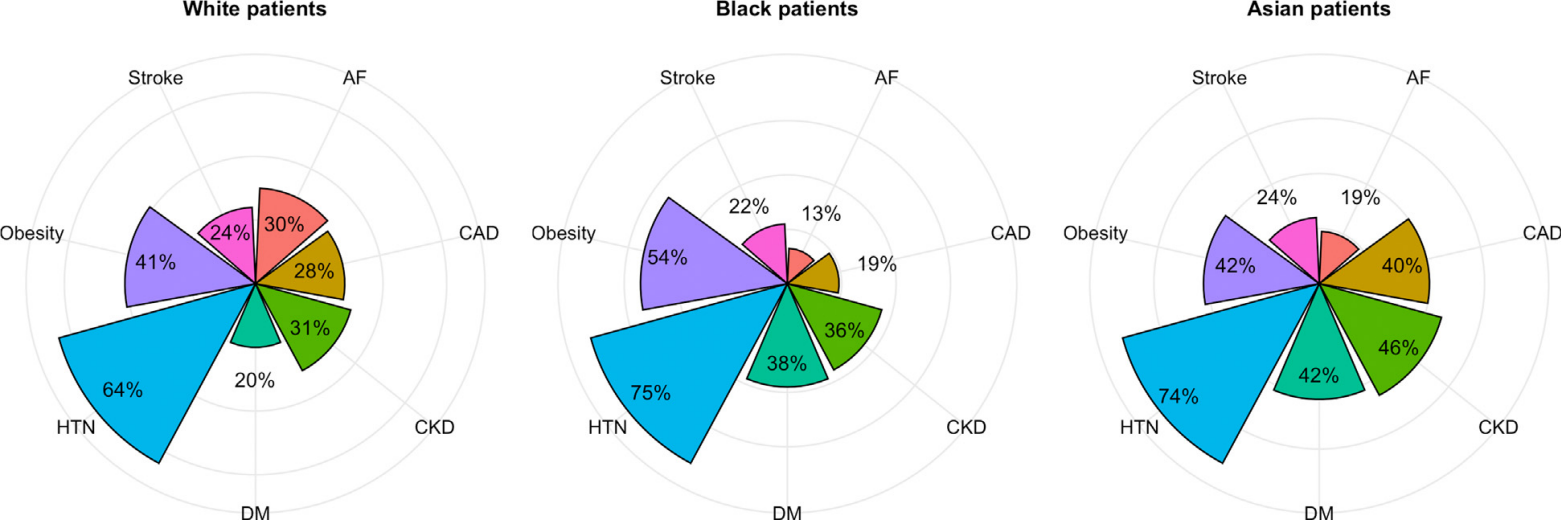
**Comorbidities at HFpEF Diagnosis** Radar plots showing the percentage of patients with each reported comorbidity mentioned prior to first HF mention, plotted separately by ethnicity. AF = atrial fibrillation; CAD = coronary artery disease; CKD = chronic kidney disease; DM = diabetes mellitus type 2; HF = heart failure; HTN = hypertension.

We identified a total of 2,223 patients with HFpEF, categorized as clinician Confirmed HFpEF (n = 231) or HFpEF diagnosed according to ESC criteria (n = 1,992) (Figure 1). Subsequently, 250 patients were excluded due to either a lack of recorded race and ethnicity (n = 147) or self-reporting as “other” (n = 96), or “mixed” (n = 7). The remaining cohort (n = 1,973) comprised 1,261 (64%) White, 578 (29%) Black, and 134 (7%) Asian patients (Figure 2). White patients were significantly older than (76 [IQR: 67-84] years) Black (71 [IQR: 56-80] years) or Asian patients (73 [IQR: 63-81] years), less likely to be female than Black patients (59% vs 64%; *P* = 0.01) and were less likely than Black patients to be in the 5th IMD quintile (21% vs 33%; *P* < 0.001) (Table 1).

**Table 1:** Baseline Patient Characteristics

		Ethnicity			
	Overall (N = 1,973)	White (n = 1,261)	Black (n = 578)	Asian (n = 134)	*P* Value^a^
Age, y	75 (63-83)	76 (67-84)	71 (56-81)	73 (63-81)	<0.001
Female	1,181 (60%)	744 (59%)	369 (64%)	68 (51%)	0.012
Lowest IMD quintile	407 (25%)	224 (21%)	162 (33%)	21 (19%)	<0.001
BMI (kg/m^2^)	29 (24-35)	28 (24-34)	31 (26-37)	28 (24-33)	<0.001
Obesity	678 (45%)	389 (41%)	245 (54%)	44 (42%)	<0.001
Diabetes mellitus	532 (27%)	256 (20%)	220 (38%)	56 (42%)	<0.001
Hypertension	1,331 (67%)	801 (64%)	431 (75%)	99 (74%)	<0.001
Atrial fibrillation	473 (24%)	375 (30%)	73 (13%)	25 (19%)	<0.001
Chronic kidney disease	663 (34%)	393 (31%)	208 (36%)	62 (46%)	<0.001
Coronary artery disease	513 (26%)	351 (28%)	109 (19%)	53 (40%)	<0.001
Stroke or TIA	458 (23%)	299 (24%)	127 (22%)	32 (24%)	0.70
Anaemia	956 (49%)	570 (45%)	305 (53%)	81 (60%)	<0.001

Values are median (IQR) or n (%).

BMI = body mass index; IMD = Index of Multiple Deprivation; TIA = transient ischemic attack.

a Kruskal-Wallis rank sum test; Pearson's chi-square test.

### Baseline characteristics

Analysis of comorbidities revealed significant differences between race and ethnicity groups (Table 1, Figure 2). Compared to White patients, Black patients had a higher BMI (31 kg/m^2^ vs 28 kg/m^2^; *P* < 0.001) and experienced higher rates of diabetes (38% vs 20%; *P* < 0.001), hypertension (75% vs 64%; *P* < 0.001), CKD (36% vs 31%; *P* = 0.04), and anemia (53% vs 45%; *P* = 0.002). Conversely, Black patients had lower rates of AF (13% vs 30%; *P* < 0.001) and CAD (19% vs 28%; *P* < 0.001). Asian patients had similar rates of obesity and stroke but were more likely to have diabetes (42% vs 20%; *P* < 0.001), CKD, and CAD (40% vs 28%; *P* < 0.001). Baseline characteristics stratified by ethnicity and diagnostic group (clinician-assigned vs NLP-identified ESC criteria patients) are available in Supplemental Table 1.

HF medication use was analyzed, showing no significant differences in loop diuretic use between ethnicities (*P* = 0.18) (Supplemental Table 2).

### HFpEF diagnostic markers

**Figure 3:**
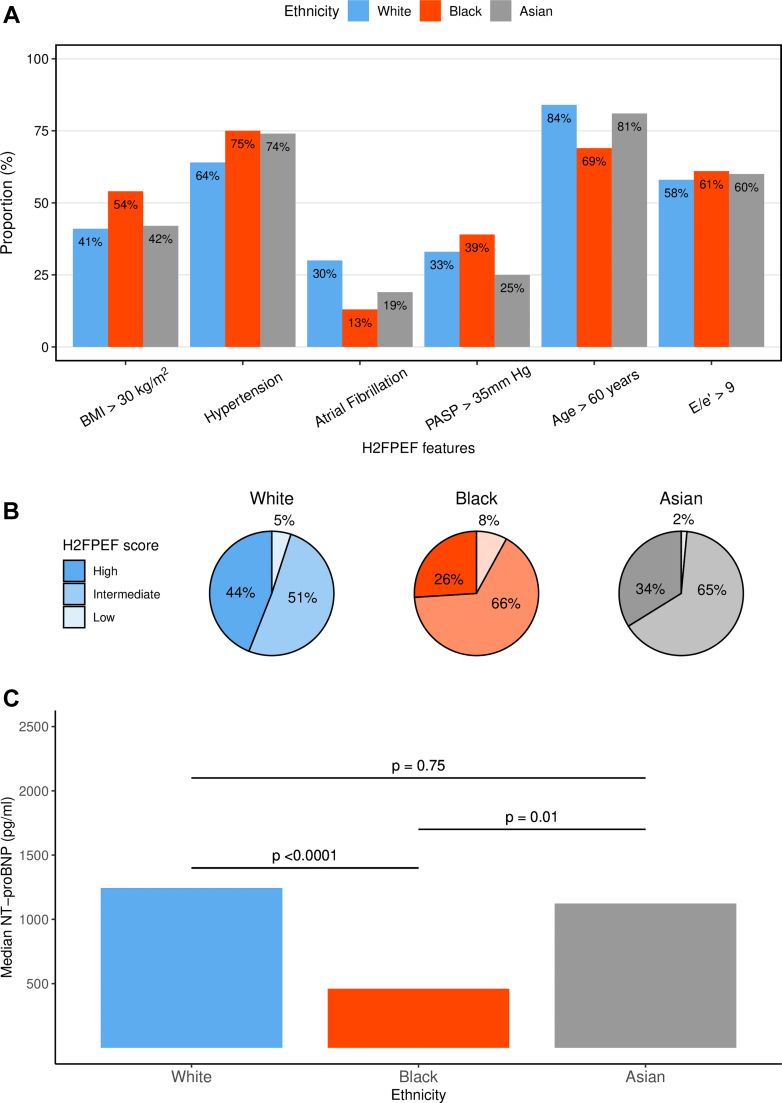
HF Markers (A) The H_2_FPEF score displayed as the percentage of patients meeting each of the score components; BMI >30 kg/m^2^, atrial fibrillation (AF), hypertension, pulmonary artery systolic pressure (PASP) > 35 mm Hg, setage >60 Years, and E/e’ ratio >9 (ratio of early diastolic mitral inflow velocity to mitral annulus relaxation velocity). Plotted as individual bars per ethnicity. (B) Pie chart depicting the percentage of patients with high (≥6), intermediate (2-5), and low (<1) H_2_FPEF scores as defined by the original publication, plotted separately by ethnicity. (C) Bar chart for median NT-proBNP stratified by ethnicity. *P* values calculated by Kruskal-Wallis Test. BMI = body mass index; HF = heart failure; NT-proBNP = N-terminal pro-B-type natriuretic peptide.

Black patients had a significantly lower baseline median NT-proBNP (460 [IQR: 127-1,634] pg/mL) compared to White (1,244 [IQR: 419-3,497] pg/mL) and Asian patients (1,123 [IQR: 288-4,051] pg/mL) (*P* < 0.001, Figure 3C). A significantly higher proportion of White patients met the ESC HF diagnostic threshold for NT-proBNP ≥125 pg/mL (or 375 pg/ml with AF) compared with Black patients (88% vs 73%, *P* < 0.001) (Table 2).

**Table 2:** Heart Failure Diagnostic Markers and Laboratory Values

		Ethnicity			
	Overall (N = 1,973)	White (n = 1,261)	Black (n = 578)	Asian (n = 134)	*P* Value^a^
NT-proBNP (pg/mL)	1,009 (286-2,914)	1,244 (419-3,497)	460 (127-1,634)	1,123 (288-4,051)	<0.001
NT-proBNP ≥125 pg/mL	621 (85%)	411 (90%)	170 (75%)	40 (87%)	<0.001
H_2_FPEF score	4.00 (3.00-6.00)	5.00 (3.00-6.00)	4.00 (3.00-6.00)	4.00 (3.00-6.00)	<0.001
H_2_FPEF score groups					<0.001
High	746 (38%)	552 (44%)	149 (26%)	45 (34%)	
Intermediate	1,111 (56%)	642 (51%)	382 (66%)	87 (65%)	
Low	116 (5.9%)	67 (5.3%)	47 (8.1%)	2 (1.5%)	

Values are median (IQR) or n (%).

NT-proBNP = N-terminal pro-B-type natriuretic peptide.

a Kruskal-Wallis rank sum test; Pearson's chi-square test.

Similarly, H_2_FPEF scores were significantly higher in White patients (median 5 [IQR: 3-6]) compared to Black patients (median 4 [IQR: 3-6]) and Asian patients (median 4 [IQR: 3-6]) (*P* < 0.001) (Table 2). Importantly, the proportion of patients meeting the diagnostic threshold (H_2_FPEF score ≥6) was significantly greater in White patients compared to Black (43.8% vs 25.8%; *P* < 0.0001) and Asian patients (43.8% vs 33.6%; *P* = 0.02) (Figure 3B). This result remained significant when restricted to patients with a clinician-assigned diagnosis of HFpEF (*P* < 0.01).

### Echocardiogram variables

The median LVEF did not differ significantly between ethnicities (*P* = 0.06) (Table 3). Indexed LV mass was significantly greater in Black patients at baseline (98 [IQR: 79-118] g/m^2^) compared with both White (90 [IQR: 75-110] g/m^2^, *P* < 0.01) and Asian patients (88 [IQR: 73-104] g/m^2^, *P* = 0.001). No significant differences were observed in diastolic dysfunction, as measured by the E/e’ ratio. However, significantly more Black patients had PASP >35 mm Hg, compared to White (39% vs 33%; *P* = 0.025) and Asian patients (39% vs 30%; *P* = 0.007). Comparing patients with high and low indexed LV mass <95/115 g/m^2^, there was no difference in the number of Black patients with H_2_FPEF ≥6 (37% vs 35%, *P* = 0.15).

**Table 3:** Baseline Echocardiography Measurements

	Ethnicity			
	White (n = 1,261)	Black (n = 578)	Asian (n = 134)	*P* Value^a^
EF				0.063
Median (IQR)	59.2 (55.6–63.9)	60.1 (55.5–65.0)	61.7 (56.6–66.0)	
Range	50.0–78.9	50.0–79.0	50.4–84.5	
LV mass index (g/m^2^)	90 (75–110)	98 (79–118)	88 (73–104)	0.004
LV mass index ≥95/115 g/m^2^	192 (38%)	124 (48%)	23 (34%)	0.013
LVEDD (cm)	4.40 (4.00–4.90)	4.40 (4.00–4.80)	4.35 (4.00–4.70)	0.5
LVPWd (cm)	1.10 (0.93–1.20)	1.20 (1.00–1.30)	1.10 (0.95–1.20)	<0.001
LVIDs (cm/m^2^)	2.87 (2.52–3.22)	2.77 (2.39–3.10)	2.73 (2.49–3.15)	0.001
LA volume (mL/m^2^)	35 (26–47)	34 (27–42)	30 (23–42)	0.20
LA volume >34 mL/m^2^	232 (62%)	109 (59%)	30 (51%)	0.20
Lateral E/e'	9.2 (6.8–13.0)	10.0 (7.2–13.6)	11.0 (8.3–14.8)	<0.001
Septal E/e'	12.7 (9.5–17.2)	12.9 (9.6–16.5)	13.8 (11.1–18.1)	0.071
Average E/e' ratio	10.0 (8.0–14.0)	11.0 (8.0–15.0)	10.0 (8.5–15.0)	0.60
E/e’ ratio >9	413 (67%)	238 (69%)	60 (77%)	0.20
TRmax (cm/s)	270 (236–308)	276 (238–319)	263 (226–296)	0.015
TRmax >280 cm/s	447 (43%)	235 (48%)	44 (37%)	0.039
PASP (mm Hg)	29 (22–38)	31 (23–41)	28 (20–35)	0.015
PASP >35 mm Hg	342 (33%)	188 (39%)	30 (25%)	0.009

Values are median (IQR) or n (%) unless otherwise indicated.

EF = ejection fraction; LA = left atrium; LVEDD = left ventricular end-diastolic diameter; LVIDs = left ventricular internal dimension at end-systole; LVPWd = left ventricular posterior wall thickness at end-diastole; PASP = pulmonary artery systolic pressure; TRmax = maximum tricuspid regurgitation velocity.

a Kruskal-Wallis rank sum test; Pearson's chi-squared test.

### Clinical outcomes

**Figure 4:**
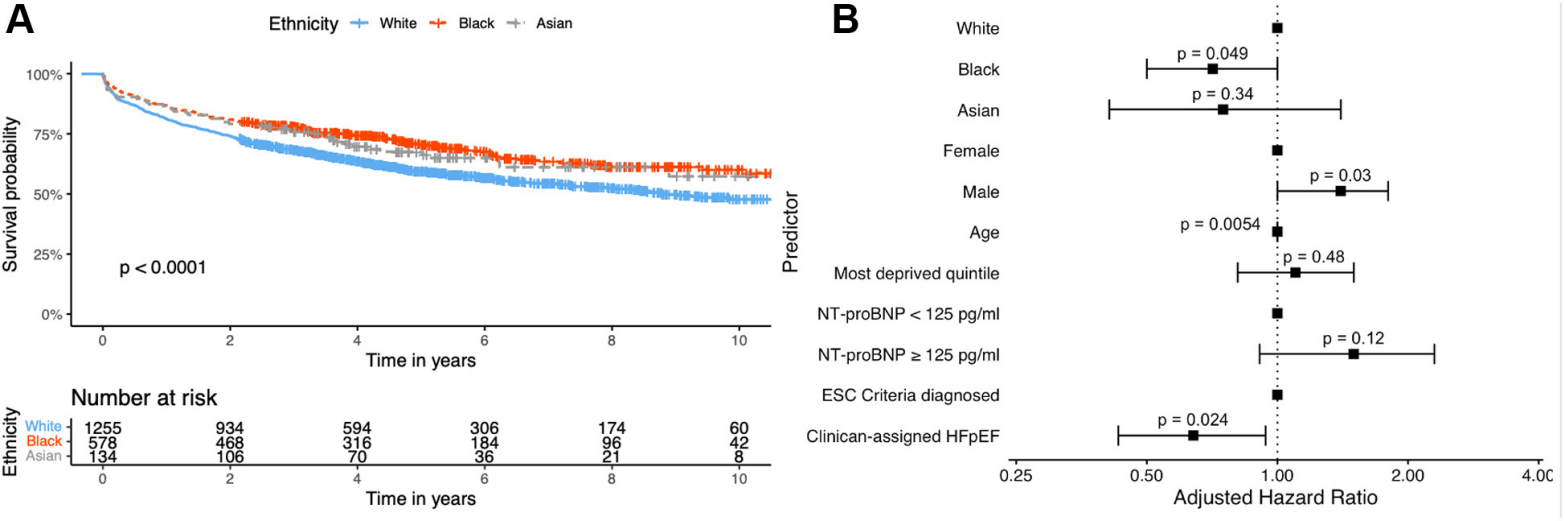
Survival Outcomes (A) Kaplan-Meier plot showing overall survival outcomes from first HF mention stratified by ethnicity and compared via log-rank test. (B) Forest plot showing adjusted hazard ratios for multivariate Cox regression analysis of overall survival from first HF mention by ethnicity, age, sex, socioeconomic deprivation (percentage in most deprived indices of multiple deprivation [IMD] quintile), NT-proBNP ≥125 pg/mL (375 pg/mL in atrial fibrillation) status and diagnostic group (clinician-assigned HFpEF or ESC criteria diagnosed). ESC = European Society of Cardiology; HF = heart failure; HFpEF = heart failure with preserved ejection fraction; NT-proBNP = N-terminal pro-B-type natriuretic peptide.

At 10 years from the first HF mention, overall survival rates were 48% (95% CI: 44%-52%) for White patients, 60% (95% CI: 55%-66%) for Black patients, and 57% (95% CI: 47%-70%) for Asian patients. White patients experienced significantly poorer survival outcomes than Black and Asian patients (*P* < 0.001) (Figure 4A). In a multivariate Cox regression analysis, adjusting for age, sex, socioeconomic deprivation, high NTproBNP levels, and diagnostic group (clinician-assigned HFpEF vs ESC diagnosed), Black patients had a lower risk of mortality compared to White patients (Figure 4B).

**Figure 5:**
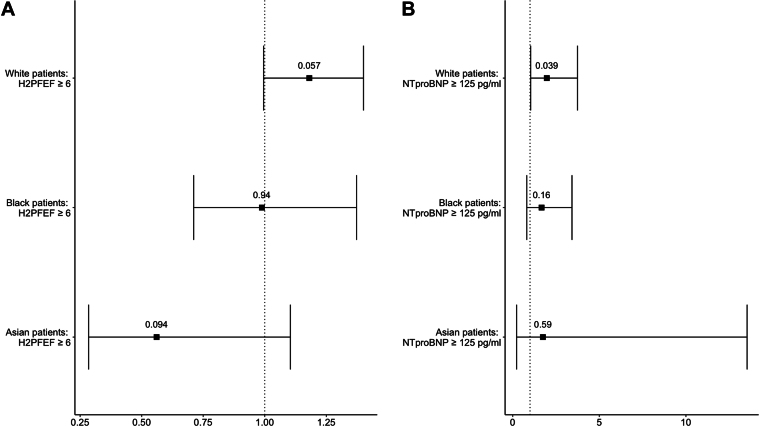
Cox Regression Analysis of HF Markers (A) Forest plot showing unadjusted hazard ratios for univariate cox regression analysis of overall survival from first HF mention by H_2_FPEF score ≥6 status stratified by ethnicity subgroup. (B) Forest plot showing unadjusted hazard ratios for univariate cox regression analysis of overall survival from first HF mention by NT-proBNP ≥125 pg/mL (375 pg/mL in atrial fibrillation) status stratified by ethnicity subgroup. HF = heart failure; NT-proBNP = N-terminal pro-B-type natriuretic peptide.

In a univariate analysis, an H_2_FPEF score ≥6 had no association with mortality in White (HR: 1.18; 95% CI: 1.00-1.41; *P* = 0.06), Black (HR: 0.99; 95% CI: 0.71-1.37; *P* > 0.9), or Asian patients (HR: 0.56; 95% CI: 0.28-1.10; *P* = 0.09) (Figure 5).

More White patients were subsequently diagnosed with AF compared to Black patients (25% vs 13%; *P* < 0.001), but not compared to Asian patients (25% vs 15%; *P* = 0.08). There was no difference in the number of patients hospitalized within the study period between White (83%), Black (82%), and Asian patients (77%) (*P* = 0.20). Equally, the number of patients with a diagnosis of stroke or transient ischemic attack after their first mention of HF was not significantly different between the ethnicities, White (24%), Black (25%), and Asian (30%) (*P* = 0.60) (Supplemental Table 3).

## Discussion

There are several important findings from this race and ethnicity analysis of HFpEF. Firstly, we demonstrated through the use of NLP to assemble a cohort of patients meeting the ESC 2021 HFpEF diagnostic criteria that the H_2_FPEF score underdiagnosed HFpEF in Black patients relative to White patients in our cohort (44% vs 26%). Secondly, we also confirmed in a diverse population that the comorbidity profile of HFpEF patients varies significantly based on ethnicity (Central Illustration). Specifically, Black patients are younger and more likely to have metabolic comorbidities, while White patients are older and had a higher prevalence of AF and CAD. Finally, White patients with HFpEF experienced higher mortality than Black patients.

**Central Illustration:**
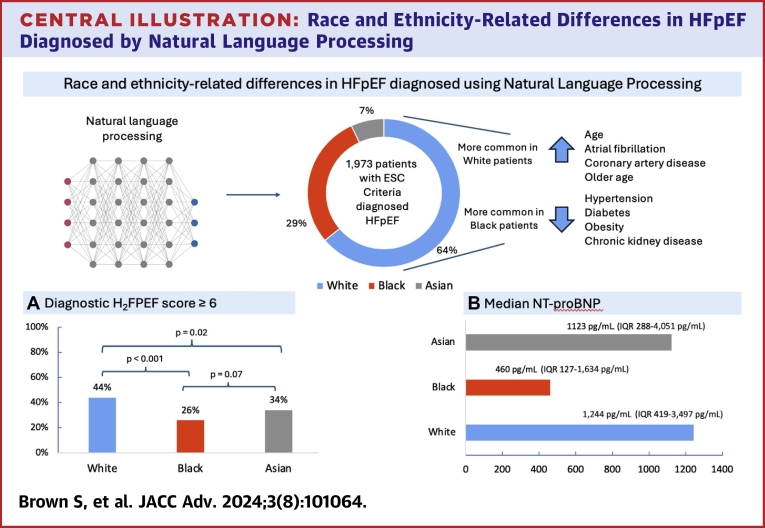
Race and Ethnicity-Related Differences in HFpEF Diagnosed by Natural Language Processing Piechart (central) shows the number of patients diagnosed with HFpEF according to the european society of cardiology diagnostic criteria stratified by self-reported ethnicity. (A) Shows a bar chart for the number of patients with H_2_FPEF score ≥6 by ethnicity, with *P* values. (B) Bar chart depicting the median NT-proBNP values for each ethnic group, along with their IQRs. ESC = European Society of Cardiology; NT-proBNP = N-terminal pro-B-type natriuretic peptide.

HFpEF is a heterogenous syndrome with distinct clinical phenotypes reflecting a varying prevalence of comorbidities.^22^ Our cohort aligns with prior descriptions, in that patients were older, predominantly female, and had a high burden of comorbidities.^12^^,^^23^, ^24^, ^25^ Importantly, we confirmed a significant interaction between the frequency of these comorbidities and ethnicity that is consistent with prior studies to date.^25^, ^26^, ^27^, ^28^, ^29^ That Black patients with HFpEF are younger and have more hypertension, obesity, and diabetes. These distinct comorbidity profiles may have pathophysiological implications that influence HFpEF development and survival. For instance, the manifestation of diabetic-predominant HFpEF in Black patients at a younger age may suggest a distinct phenotype driven more by systemic inflammation than other forms of HFpEF.^30^, ^31^, ^32^

Beyond pathophysiology, these findings have important diagnostic implications. We found that in a HFpEF cohort selected using the ESC diagnostic criteria, Black and Asian patients had statistically significantly lower H_2_FPEF scores and were less likely to be categorized as likely HFpEF (ie, H_2_FPEF ≥6). This disconnect between the ESC criteria, which is based on biochemistry and echocardiography values, and the H_2_FPEF score is likely due to the greater emphasis on comorbidities in the H_2_FPEF scoring system, particularly AF, as has been noted in other cohorts.^33^ The weighting given to AF in H_2_FPEF (the presence of paroxysmal or persistent AF scoring three out of nine possible points) becomes highly relevant in groups with low rates of AF, such as Black patients in our HFpEF cohort (only 13% compared to an overall prevalence of 24% in our HFpEF population and 34% in the original H_2_FPEF HFpEF cohort) (Figures 2 and 3A).^7^ This may explain why H_2_FPEF has limited diagnostic ability in our Black patient cohort (Figure 3B). Previous studies also demonstrated variance in the sensitivity of H_2_FPEF in diagnosing HFpEF dependent on the background rate of AF, with H_2_FPEF performing the poorest in populations of younger patients with less AF and better in the opposite groups.^8^^,^^34^ We note that our findings were consistent even when considering only those patients with a clinician-assigned formal diagnosis of HFpEF and so cannot be solely attributed to differences between the populations captured by the ESC criteria and H_2_FPEF scores. The reduced clinical applicability in diagnosing Black patients with HFpEF, who already have lower NT-proBNP values,^35^ has consequences for trial recruitment and the treatment. Especially now SGLT2i are guideline-recommended therapy.^36^

The diagnosis of HFpEF continues to be a challenge. Each diagnostic algorithm or criteria has a different focus, incorporates different variables and thus may preferentially diagnose different HFpEF phenotypes. The ESC criteria acknowledge this and are purposefully broad to enhance their applicability, particularly when other diagnostic aids demonstrate consistent discordance in their categorization of HFpEF and vary widely in their sensitivities.^6^^,^^8^^,^^21^^,^^33^^,^^37^ This highlights the issue of currently having one HFpEF definition that may encompass a variety of phenotypes that differ by ethnicity and prognosis. We used the current gold standard ESC criteria with its broader applicability, but future research will need to address this diagnostic uncertainty and find ways to understand HFpEF better for each individual. Developing a more comprehensive understanding of the diagnosis of HFpEF across race and ethnicity should lead to better health equity by improving clinical trial diversity. In the meantime, clinicians should be cautious as to the potential pitfalls on relying on a single scoring system to diagnose HFpEF in Black patients and instead use them in conjunction with other clinical and laboratory findings to reach an accurate diagnosis.

We found that Black patients in our cohort had a lower mortality compared with White patients. This has been demonstrated elsewhere by other groups,^25^ even when adjusting for comorbidities.^26^ In our cohort, when adjusted for factors including age and high NT-proBNP levels, the difference in survival lost significance suggesting that the reason for the lower mortality relates to HFpEF being less advanced in these patients.

Strengths of our study include the use of NLP AI methods for case identification, a technique that has been validated previously by our group,^14^ and others.^38^ Furthermore, by applying the ESC HFpEF diagnostic criteria as our inclusion criteria, we ensured that our patients met an internationally recognized definition of HFpEF. Additionally, collation of a cohort of this type would traditionally be performed by manual review, which itself is an imperfect process, difficult to standardized and open to personal biases. The review of over 1 million patients would not have been possible manually and underlines the utility of our approach using NLP. Finally, by being performed in the National Health Service, where health care is free at the point of use, this study is likely less impacted by systemic differences such as payer biases.

### Study Limitations

Firstly, this was a single-center retrospective study that relied on accurate free-text clinical documentation. Additionally, we were unable to obtain the cause of death so could not explore differences between cardiac and noncardiac death. Most importantly, we acknowledge that race and ethnicity are social constructs without scientific meaning and thus the grouping of race and ethnicity should be done via self-identification using the broadest possible language. While certain groups display disproportionate burdens of disease, a large proportion of this may reflect systemic disparities in the social determinants of health. A limitation, therefore, is our lack of ability to subcategorize race and ethnicities, which risks the oversimplification of findings attributable to one race or ethnicity.

## Conclusions

HFpEF is a diverse clinical syndrome requiring ongoing efforts to identify clinical and pathophysiological subgroups to improve our understanding and treatment options. Leveraging an NLP-based AI approach to quantify health inequities in HFpEF management, we discovered that established markers systematically underdiagnose HFpEF in Black patients, likely due to differences in the underlying comorbidity patterns. Clinicians should be aware of these limitations and its implications for future research, trial recruitment, and treatment.

Perspectives **COMPETENCY IN MEDICAL KNOWLEDGE:** The under-diagnosis of HFpEF is a clinical problem, as evidenced by our recent AI-based cohort study.^14^ This new study suggests there is an enhanced issue in Black patients with HFpEF, as predictive diagnostic systems may be biased in favour of the comorbidity patterns more prevalent in White patients with HFpEF.

**TRANSLATIONAL OUTLOOK:** HFpEF is a heterogeneous syndrome and we have demonstrated that there is a need for improved ways of diagnosing HFpEF that are validated across diverse cohorts. Clinicians should therefore be aware of the limitations of current scoring systems. Future studies should focus on using AI to better phenotype HFpEF across ethnicities and develop equitable multimodality diagnostic tools.

## Funding support and author disclosures

Professor Shah has served as an advisor to Forcefield Therapeutics and CYTE–Global Network for Clinical Research. Professor McDonagh has received speaker fees or advisory board fees from Abbott, Edwards, Boehringer Ingelheim, and AstraZeneca. This work was supported by grants from the 10.13039/501100000274British Heart Foundation (CH/1999001/11735, RG/20/3/34823, and RE/18/2/34213 to Professor Shah; CC/22/250022 to Dr Dobson, Professor Shah, Dr Teo, and Dr Gallagher) and King’s College Hospital Charity (D3003/122022/Shah/1188 to Dr Shah). Dr Gallagher and Dr Bromage are each supported by MRC Clinician Scientist Fellowships (MR/Y001311/1 to Dr Gallagher, MR/X001881/1 to Dr Bromage). All other authors have reported that they have no relationships relevant to the contents of this paper to disclose.

## References

[1] Borlaug B.A., Sharma K., Shah S.J., Ho J.E. (2023). Heart failure with preserved ejection fraction. J Am Coll Cardiol.

[2] Owan T.E., Hodge D.O., Herges R.M., Jacobsen S.J., Roger V.L., Redfield M.M. (2006). Trends in prevalence and outcome of heart failure with preserved ejection fraction. N Engl J Med.

[3] Chang P.P., Wruck L.M., Shahar E. (2018). Trends in Hospitalizations and survival of acute Decompensated heart failure in Four US Communities (2005–2014). Circulation.

[4] Sueta D., Yamamoto E., Nishihara T. (2019). H2FPEF score as a prognostic value in HFpEF patients. Am J Hypertens.

[5] Verbrugge F.H., Reddy Y.N.V., Sorimachi H., Omote K., Carter R.E., Borlaug B.A. (2021). Diagnostic scores predict morbidity and mortality in patients hospitalized for heart failure with preserved ejection fraction. Eur J Heart Fail.

[6] Selvaraj S., Myhre P.L., Vaduganathan M. (2020). Application of diagnostic algorithms for heart failure with preserved ejection fraction to the Community. JACC Heart Fail.

[7] Reddy Y.N.V., Carter R.E., Obokata M., Redfield M.M., Borlaug B.A. (2018). A Simple, evidence-based approach to Help Guide diagnosis of heart failure with preserved ejection fraction. Circulation.

[8] Ouwerkerk W., Tromp J., Jin X. (2020). Heart failure with preserved ejection fraction diagnostic scores in an Asian population. Eur J Heart Fail.

[9] Solomon S.D., McMurray J.J.V., Claggett B. (2022). Dapagliflozin in heart failure with Mildly reduced or preserved ejection fraction. N Engl J Med.

[10] Anker S.D., Butler J., Filippatos G. (2021). Empagliflozin in heart failure with a preserved ejection fraction. N Engl J Med.

[11] Tahhan A.S., Vaduganathan M., Greene S.J. (2018). Enrollment of older patients, Women, and racial and ethnic Minorities in Contemporary heart failure clinical trials. JAMA Cardiol.

[12] Uijl A., Savarese G., Vaartjes I. (2021). Identification of distinct phenotypic clusters in heart failure with preserved ejection fraction. Eur J Heart Fail.

[13] McDonagh T.A., Metra M., Adamo M. (2021). 2021 ESC Guidelines for the diagnosis and treatment of acute and chronic heart failure. Eur Heart J.

[14] Wu J., Biswas D., Ryan M. (2024). Artificial intelligence methods for improved detection of undiagnosed heart failure with preserved ejection fraction (HFpEF). Eur J Heart Fail.

[15] Farajidavar N., O’Gallagher K., Bean D. (2022). Diagnostic signature for heart failure with preserved ejection fraction (HFpEF): a machine learning approach using multi-modality electronic health record data. BMC Cardiovasc Disord.

[16] Kraljevic Z., Searle T., Shek A. (2021). Multi-domain clinical Natural Language Processing with MedCAT: the medical concept annotation Toolkit. Artif Intell Med.

[17] Searle T., Kraljevic Z., Bendayan R., Bean D., Dobson R. (2019). Proceedings of the 2019 Conference on Empirical methods in Natural Language Processing and the 9th international Joint Conference on Natural Language Processing (EMNLP-IJCNLP): system Demonstrations.

[18] Bean D.M., Kraljevic Z., Searle T. (2020). Angiotensin-converting enzyme inhibitors and angiotensin II receptor blockers are not associated with severe COVID-19 infection in a multi-site UK acute hospital trust. Eur J Heart Fail.

[19] Flanagin A., Frey T., Christiansen S.L. (2021). Updated guidance on the reporting of race and ethnicity in medical and science Journals. JAMA.

[20] English indices of deprivation 2019 - GOV.UK. https://www.gov.uk/government/statistics/english-indices-of-deprivation-2019.

[21] Sepehrvand N., Alemayehu W., Dyck G.J.B. (2019). External validation of the H2F-PEF Model in diagnosing patients with heart failure and preserved ejection fraction. Circulation.

[22] Redfield M.M., Borlaug B.A. (2023). Heart failure with preserved ejection fraction. JAMA.

[23] Schrub F., Oger E., Bidaut A. (2020). Heart failure with preserved ejection fraction: a clustering approach to a heterogenous syndrome. Arch Cardiovasc Dis.

[24] Segar M.W., Patel K.V., Ayers C. (2020). Phenomapping of patients with heart failure with preserved ejection fraction using machine learning-based unsupervised cluster analysis. Eur J Heart Fail.

[25] Ziaeian B., Heidenreich P.A., Xu H. (2017). Race/ethnic differences in outcomes among hospitalized Medicare patients with heart failure and preserved ejection fraction. JACC Heart Fail.

[26] Sharma K., Mok Y., Kwak L. (2020). Predictors of mortality by sex and race in heart failure with preserved ejection fraction: ARIC Community Surveillance study. J Am Heart Assoc.

[27] Gupta D.K., Shah A.M., Castagno D. (2013). Heart failure with preserved ejection fraction in African Americans. JACC Heart Fail.

[28] Goyal P., Paul T., Almarzooq Z.I. (2017). Sex- and race-related differences in characteristics and outcomes of Hospitalizations for heart failure with preserved ejection fraction. J Am Heart Assoc.

[29] Cohen J.B., Schrauben S.J., Zhao L. (2020). Clinical Phenogroups in heart failure with preserved ejection fraction. JACC Heart Fail.

[30] Paulus W.J., Zile M.R. (2021). From systemic inflammation to myocardial Fibrosis. Circ Res.

[31] McHugh K., DeVore A.D., Wu J. (2019). Heart failure with preserved ejection fraction and diabetes. J Am Coll Cardiol.

[32] Shah S.J., Kitzman D.W., Borlaug B.A. (2016). Phenotype-specific treatment of heart failure with preserved ejection fraction. Circulation.

[33] Nikorowitsch J., Bei der Kellen R., Kirchhof P. (2021). Applying the ESC 2016, H2FPEF, and HFA-PEFF diagnostic algorithms for heart failure with preserved ejection fraction to the general population. ESC Heart Fail.

[34] Tada A., Nagai T., Omote K. (2021). Performance of the H2FPEF and the HFA-PEFF scores for the diagnosis of heart failure with preserved ejection fraction in Japanese patients: a report from the Japanese multicenter registry. Int J Cardiol.

[35] Gupta D.K., de Lemos J.A., Ayers C.R., Berry J.D., Wang T.J. (2015). Racial differences in Natriuretic Peptide levels. JACC Heart Fail.

[36] McDonagh T.A., Metra M., Adamo M. (2024). 2023 Focused Update of the 2021 ESC Guidelines for the diagnosis and treatment of acute and chronic heart failure. Eur J Heart Fail.

[37] Sanders-van Wijk S., Barandiarán Aizpurua A., Brunner-La Rocca H.P. (2021). The HFA-PEFF and H2FPEF scores largely disagree in classifying patients with suspected heart failure with preserved ejection fraction. Eur J Heart Fail.

[38] Cunningham J.W., Singh P., Reeder C. (2023). Natural Language processing for Adjudication of heart failure in a multicenter clinical trial. JAMA Cardiol.

