# Supplementary material for: Race- and Ethnicity-Related Differences in Heart Failure With Preserved Ejection Fraction Using Natural Language Processing
Source: JACC Adv. 2024 Jul 2;3(8):101064. doi: 10.1016/j.jacadv.2024.101064 (PMC11268103; doi:10.1016/j.jacadv.2024.101064)
Supplement: Supplemental Data [file mmc1.docx]

Supplemental Table 1. Patient Characteristics stratified by ethnicity and diagnostic group

|  | **White** | | **Black** | | **Asian** | |
| --- | --- | --- | --- | --- | --- | --- |
| **Characteristic***^a^* | **Confirmed HFpEF**, N = 122*^b^* | **ESC Criteria**, N = 1,139*^b^* | **Confirmed HFpEF**, N = 65*^b^* | **ESC Criteria**, N = 513*^b^* | **Confirmed HFpEF**, N = 17*^b^* | **ESC Criteria**, N = 117*^b^* |
| Age | 81 (74, 86) | 75 (66, 84) | 80 (72, 84) | 69 (55, 80) | 80 (76, 82) | 72 (63, 79) |
| Female Sex | 84 (69%) | 660 (58%) | 42 (65%) | 327 (64%) | 7 (41%) | 61 (52%) |
| Lowest IMD quintile | 28 (27%) | 196 (21%) | 15 (25%) | 147 (34%) | 2 (12%) | 19 (21%) |
| BMI (kg/m2) | 29 (25, 37) | 28 (24, 34) | 32 (27, 37) | 31 (26, 37) | 28.4 (26.2, 31.4) | 27.9 (24.2, 33.7) |
| Obesity | 55 (47%) | 334 (40%) | 37 (64%) | 208 (53%) | 6 (38%) | 38 (43%) |
| Diabetes Mellitus | 26 (21%) | 230 (20%) | 31 (48%) | 189 (37%) | 9 (53%) | 47 (40%) |
| Hypertension | 99 (81%) | 702 (62%) | 60 (92%) | 371 (72%) | 16 (94%) | 83 (71%) |
| Atrial Fibrillation | 60 (49%) | 315 (28%) | 13 (20%) | 60 (12%) | 8 (47%) | 17 (15%) |
| Chronic Kidney Disease | 48 (39%) | 345 (30%) | 30 (46%) | 178 (35%) | 13 (76%) | 49 (42%) |
| Coronary Artery Disease | 40 (33%) | 311 (27%) | 16 (25%) | 93 (18%) | 12 (71%) | 41 (35%) |
| Stroke or TIA | 46 (38%) | 253 (22%) | 21 (32%) | 106 (21%) | 5 (29%) | 27 (23%) |
| Anaemia | 36 (30%) | 534 (47%) | 34 (53%) | 271 (53%) | 9 (53%) | 72 (62%) |
| NT-proBNP (pg/ml) | 1,460 (696, 3,230) | 1,200 (349, 3,632) | 558 (198, 1,647) | 414 (121, 1,602) | 2,351 (785, 5,181) | 927 (249, 3,084) |
| H2FPEF score |  |  |  |  |  |  |
| High | 89 (73%) | 463 (41%) | 30 (46%) | 119 (23%) | 11 (65%) | 34 (29%) |
| Intermediate | 32 (26%) | 610 (54%) | 35 (54%) | 347 (68%) | 6 (35%) | 81 (69%) |
| Low | 1 (0.8%) | 66 (5.8%) | 0 (0%) | 47 (9.2%) | 0 (0%) | 2 (1.7%) |
| *^a^*Abbreviations: IMD = Index of Multiple Deprivation, TIA = Transient Ischaemic Attack | | | | | | |
| *^b^*Median (IQR); n (%) | | | | | | |

Supplemental Table 2. Medication Use

|  | **Ethnicity** | | |  |
| --- | --- | --- | --- | --- |
| **Characteristic** | **White**, N = 1,261*^1^* | **Black**, N = 578*^1^* | **Asian**, N = 134*^1^* | **p-value***^2^* |
| Loop diuretics | 996 (79%) | 443 (77%) | 112 (84%) | 0.2 |
| ACEi or ARBs | 795 (63%) | 400 (69%) | 105 (78%) | **<0.001** |
| Beta blockers | 754 (60%) | 336 (58%) | 97 (72%) | **0.009** |
| Calcium Channel Blockers | 576 (46%) | 407 (70%) | 85 (63%) | **<0.001** |
| SGLT2 inhibitors | 19 (1.5%) | 8 (1.4%) | 1 (0.7%) | >0.9 |
| Statins | 613 (49%) | 317 (55%) | 77 (57%) | **0.014** |
| Antiplatelets | 754 (60%) | 344 (60%) | 100 (75%) | **0.003** |
| Anticoagulants | 710 (56%) | 231 (40%) | 56 (42%) | **<0.001** |
| Amiodarone | 114 (9.0%) | 29 (5.0%) | 10 (7.5%) | **0.011** |
| Sotalol | 24 (1.9%) | 5 (0.9%) | 2 (1.5%) | 0.2 |
| Insulin | 291 (23%) | 241 (42%) | 57 (43%) | **<0.001** |
| *^1^*n (%) | | | | |
| *^2^*Pearson's Chi-squared test; Fisher's exact test | | | | |

Supplemental Table 3. Clinical Outcomes including stroke, atrial fibrillation and hospitalisation

|  | **Ethnicity** | | |  |
| --- | --- | --- | --- | --- |
| **Outcome***^1^* | **White**, N = 1,261 | **Black**, N = 578 | **Asian**, N = 134 | **p-value***^2^* |
| Stroke or TIA, n (%) | 297 (24%) | 147 (25%) | 30 (22%) | 0.6 |
| New AF, n (%) | 226 (18%) | 64 (11%) | 16 (12%) | **<0.001** |
| All hospitalisations, n (%) | 1,046 (83%) | 475 (82%) | 103 (77%) | 0.2 |
| *^1^*TIA = Transient Ischaemic Attack | | | | |
| *^2^*Pearson's Chi-squared test | | | | |
